# Optimizing Antitumor Effect of Triple-Negative Breast Cancer via Rosmarinic Acid–β-Cyclodextrin Inclusion Complex

**DOI:** 10.3390/pharmaceutics16111408

**Published:** 2024-11-01

**Authors:** Yuan Li, Muhammad Inam, Muhammad Waqqas Hasan, Kaixin Chen, Zhongqian Zhang, Yongcheng Zhu, Jiayu Huang, Zhuowen Wu, Wenjie Chen, Min Li

**Affiliations:** 1 Department of Emergency, The Second Affiliated Hospital, Guangzhou Medical University, Guangzhou 510260, China; 2022211519@stu.gzhmu.edu.cn (Y.L.); 2022390020@gzhmu.edu.cn (M.I.); waqqashasan@gzhmu.edu.cn (M.W.H.); 2023211658@stu.gzhmu.edu.cn (Z.Z.); 2012690591@gzhmu.edu.cn (Y.Z.); jiayuhuang1@stu.gzhmu.edu.cn (J.H.); wuzhuowen2807@gzhmu.edu.cn (Z.W.); 2 Guangdong Province & NMPA & State Key Laboratory, School of Pharmaceutical Sciences, Guangzhou Medical University, Guangzhou 511436, China; 3 Medical Science and Technology Innovation Center, School of Chemistry and Pharmaceutical Engineering, Shandong First Medical University and Shandong Academy of Medical Sciences, Jinan 250117, China; 4 Graduate School of Biomedical Engineering, ARC Centre of Excellence in Nanoscale Biophotonics, Faculty of Engineering, UNSW Sydney, Sydney, NSW 2052, Australia; z5604048@ad.unsw.edu.au; 5 Sydney Vital Translational Cancer Research Centre, Westbourne St., Sydney, NSW 2065, Australia

**Keywords:** rosmarinic acid, cyclodextrin, physicochemical, molecular docking, inclusion complex, TNBC, anticancer

## Abstract

**Background**: Rosmarinic acid (ROS) has gained notable attention for its anticancer potential; however, its limited aqueous solubility hinders its effective delivery and application in pharmaceutical formulations. **Methods**: To overcome this limitation, an inclusion complex of ROS with β-cyclodextrin (β-CD) was prepared using the recrystallization method. The resultant ROS–β-CD complex was comprehensively characterized by powder X-ray diffraction (PXRD), differential scanning calorimetry (DSC), Fourier transform infrared spectroscopy (FT-IR), and scanning electron microscopy (SEM). **Results:** The ROS–β-CD complex showed a significant improvement in the solubility and dissolution profile of ROS, underscoring its potential for enhanced bioavailability and therapeutic efficacy in pharmaceutical applications. In vitro assays were performed to assess the effects on cell viability, proliferation, apoptotic pathways, and 3D spheroid tumor models. **Conclusions:** The results demonstrated that ROS–β-CD exhibited superior anticancer properties compared to free ROS, effectively reducing the viability and proliferation of the MD-MBA-231 cell line and inducing apoptosis. This research signifies a substantial advancement in developing therapeutic strategies for TNBC, leveraging the distinct properties of the ROS–β-CD inclusion complex.

## 1. Introduction

The solubility of orally administered drugs is critical in pharmaceutical development and therapeutic efficacy [1]. The dissolution capacity of a drug within the gastrointestinal tract significantly influences its bioavailability, representing the portion of the administered dose that influences the systemic circulation and delivers a therapeutic impact [2]. Challenges arise with poorly soluble drugs, hindering their absorption and reducing bioavailability, consequently affecting therapeutic results. Formulation design addresses these challenges by prioritizing strategies to enhance the solubility of oral drugs [3,4,5]. Approaches such as incorporating solubilizing agents, reducing particle size, and designing drug delivery systems are employed to improve solubility and bioavailability [6,7]. Efficient solubility is pivotal in ensuring a higher proportion of the administered drug is absorbed, thereby optimizing therapeutic effects and contributing to the overall success of oral drug formulations in clinical applications [2,8,9].

Triple-negative breast cancer (TNBC) is a subtype of breast cancer defined by the absence of estrogen receptors (ERs), progesterone receptors (PRs), and human epidermal growth factor receptor 2 (HER2). TNBC comprises approximately 10–15% of breast cancer cases and is characterized by the absence of estrogen and progesterone receptors, rendering it particularly aggressive. Distinguished by its rapid growth and dissemination, TNBC presents challenges in treatment, with fewer therapeutic paths and a generally poor prognosis compared to other invasive breast cancer subtypes [10]. Moreover, TNBC often acquires resistance to standard chemotherapy following repeated exposure to primary treatment agents. Therefore, the imperative is the development of tailored formulations to surmount these challenges in TNBC therapy [11]. Its aggressive nature, limited treatment options, and high recurrence rates further complicate management. Current research efforts are concentrated on addressing these challenges by developing new therapeutic approaches and enhancing patient outcomes.

Rosmarinic acid (ROS), a natural polyphenolic compound, has garnered attention for its potential as an anticancer agent [12]. Studies indicate that it demonstrates anticancer properties by modulating signaling pathways involved in cancer cell growth [13,14]. Nevertheless, a challenge in utilizing ROS for cancer therapy lies in its limited water solubility [15]. This drawback can impact its bioavailability, absorption, and overall efficacy. To address this issue, diverse strategies such as formulation techniques to enhance the water solubility of ROS are important. Overcoming these solubility challenges is essential for solving the complete therapeutic potential of ROS for cancer treatment [16].

The formation of inclusion complexes, particularly with cyclodextrins (CDs), reveals an erudite key to the bioavailability challenge. β-CD, characterized by its cyclic structure, exhibits a distinctive ability to encapsulate hydrophobic entities, enriching their solubility, dissolution, and stability [17,18,19,20]. A reflective understanding of the physicochemical intricacies characteristic of the inclusion complex is pivotal for unraveling its potential as a delivery system capable of surmounting the limitations characteristic of conventional formulations [21,22]. Physicochemical analysis of the ROS–β-CD inclusion complex assumes paramount significance in evaluating its solubility and dissolution profiles. By encapsulating ROS within the hydrophobic cavity of β-CD, the inclusion complex not only addresses solubility but also may protect the ROS from degradation, ensuring a more sustained and controlled release. The inclusion complex, stimulated by its enhanced solubility and stability, can potentially elevate drug delivery accuracy to the target site. This study endeavors to unravel the intricate relationship between the physicochemical attributes of the ROS–β-CD inclusion complex and its propensity to optimize drug delivery, with a specific focus on TNBC cells. This research explores the physicochemical properties of the inclusion complex, aiming to enhance ROS’s therapeutic potential against breast cancer by improving solubility, stability, and controlled drug release. The study offers a novel approach to overcoming bioavailability challenges, potentially advancing breast cancer treatment [23].

Numerous studies on ROS complexes have been conducted, exploring diverse applications such as ROS inclusion complexes focusing on improving solubility, stability, and antioxidant activity for food applications [24], as well as spectroscopic characterization and antioxidant properties of ROS inclusion complexes with natural and derivative cyclodextrins [25]. Furthermore, inclusion complexes of ROS with β-CD have been examined using stoichiometry, association constants, and antioxidant potential [26]. Our study advances this field by investigating the antitumor efficacy of rosmarinic acid against triple-negative breast cancer (TNBC) cells. We employed β-CD to enhance drug delivery and used docking molecular modeling to obtain detailed insights into molecular interactions. The novelty of our work lies in its targeted medical application for TNBC treatment and the integration of advanced molecular modeling techniques. This distinguishes our study from those focused-on food science or general antioxidant properties, contributing significantly to the field.

## 2. Materials and Methods

### 2.1. Materials

**Scheme 1:**
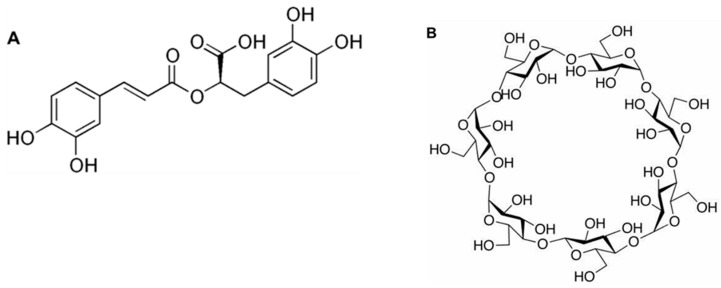
The chemical structure of (**A**) ROS and (**B**) β-CD.

Rosmarinic acid (C_18_H_16_O_8_) and β-cyclodextrin (C_42_H_70_O_35_) of 99% purity (Scheme 1) were purchased from Shanghai Macklin Biochemical Co. Ltd. (Shanghai, China). The MD-MBA-231 breast cancer cell line was purchased from the Cell Bank, Chinese Academy of Sciences (Shanghai, China). The DMEM cell culture medium for MD-MBA-231 breast cancer cells was purchased from Gibco Ltd. (Billings, MT, USA). All the other required materials, solvents, and chemicals were purchased from commercial sources and used upon receipt.

### 2.2. Preparation of Inclusion Complex

The inclusion complex of ROS with β-CD was synthesized through the recrystallization method, as previously reported [27,28]. In brief, an equal molar ratio of ROS (0.5 mmol, 0.180 g) and β-CD (0.5 mmol, 0.567 g) was added to a 20 mL mixture of EtOH-H_2_O (7:3 *v*/*v*), refluxed, and stirred for 24 h at 80 °C. The resulting solution was cooled to room temperature. The fine white candy-shaped crystalline form of the ROS–β-CD inclusion complex was obtained upon slow evaporation.

### 2.3. Solid-State Characterizations

The powder X-ray diffraction (PXRD) patterns of ROS, β-CD, and ROS–β-CD were recorded using a Rigaku D/Max-2550PC instrument (Tokyo, Japan). The system utilized a rotating anode Cu-target X-ray source with a wavelength (λ) of 1.5406 Å, operating at 40 kV and 250 mA. The scanning range was set from 3.0° to 90° with a speed of 5° per minute and an incremental step size of 0.02° over 0.5–3 s. Fourier transform infrared (FT-IR) spectra were acquired using a Thermo Scientific Nicolet iS50 FT-IR spectrometer (Thermo Fisher Scientific Co., Ltd., Waltham, MA, USA) in the 400–4000 cm⁻¹ range, employing potassium bromide (KBr) diffuse reflectance mode. Approximately 2 mg of the sample was mixed with 100 mg of KBr, manually blended in a mortar, and compacted into thin pellets. The morphology of the ROS–β-CD inclusion complex was examined using a scanning electron microscopy (SEM; Sigma 500, Zeiss, Jena, Germany) at an accelerating voltage of 4 kV without any coating. For differential scanning calorimetry (DSC) analysis, a TA DSCQ100 instrument (TA Instruments, New Castle, Germany) was used. The DSC analysis was carried out under a nitrogen atmosphere (nitrogen flow rate of 50 cm^3^/min) to maintain an inert environment and prevent sample oxidation. Calibration was performed using indium and zinc as standards to ensure measurement accuracy. Samples were placed (4 to 7 mg) in standard aluminum pans, and the analysis was conducted at atmospheric pressure, scanning a temperature range from 35 °C to 500 °C with a calibrated temperature accuracy of 0.02 °C.

### 2.4. Solubility and Dissolution Rate

Solubility and dissolution studies of ROS and the ROS–β-CD inclusion complex in water at pH 7, phosphate-buffered solution 6.8 and acidic medium pH 1.2 were performed using a Thermo Logical Advancement UV-vis spectrometer at a controlled 37 °C temperature [29,30]. The experiments began by deliberately achieving supersaturation of the solutions, which were then continuously stirred at 500 rpm using a magnetic stirrer for 24 h at the specified temperature. Subsequently, the suspension was filtered through a Whatman 0.45 µm syringe filter and appropriately diluted. Concentrations were determined using a standard curve based on absorbance measurements (within the 200 to 800 nm wavelength range (Supplementary Figure S1) at a maximum wavelength (λmax) of 330 nm). For the dissolution rate assessment, samples (ROS and ROS–β-CD) were placed into 500 mL media (pH 7, pH 6.8 and pH 1.2) and stirred at 150 rpm for 150 min, maintaining the temperature at 37 °C. At predefined intervals, 3 mL of the dissolution medium was withdrawn and replaced with an equivalent volume of fresh medium to maintain a consistent volume. The absorbance at λmax was recorded for each withdrawn solution, and the concentrations were calculated using the standard curve. The identity and stability of the remaining samples were further confirmed by PXRD (Supplementary Figures S2 and S3).

### 2.5. Phase Solubility

Phase solubility was investigated using the Thermo Logical Advancement UV-vis spectrometer [31]. Distilled water with a pH of 7.0 served as the solvent, and various concentrations (2–20 mM) of β-CD were prepared. An excess amount of ROS (50 mg) was added to each concentration, which was then equilibrated for 24 h at 37 °C with continuous stirring at 500 rpm. Following equilibration, the solubility of the compound in each solution was determined by measuring absorbance at 330 nm (λ_max_) using a standard curve constructed from the different concentrations of ROS. The resulting data were analyzed to reveal solubility patterns and potential complexation behavior between the ROS and β-CD. The apparent binding constant (Kc) and complexation efficiency (CE) were determined from the slope of the phase solubility curve, using the following equation:

$$Kc=\frac{slope}{S₀(1-slope)}$$

where S_0_ is the solubility of ROS in water in the absence of β-CD.

$$CE=\frac{slope}{1-slope}$$

### 2.6. Molecular Docking Analysis of ROS with Bcl-2 Protein

The Bcl-2 protein is pivotal in breast cancer due to its regulatory role in apoptosis or programmed cell death. As an anti-apoptotic protein, Bcl-2 inhibits apoptosis, promoting cell survival. Under physiological conditions, Bcl-2 maintains a critical balance between cell survival and apoptosis, facilitating the removal of damaged or superfluous cells. To investigate ROS-mediated apoptosis in breast cancer, molecular docking studies were performed. The molecular docking technique is used to elucidate the interaction between ROS and the anti-apoptotic Bcl-2 protein. The three-dimensional structure of Bcl-2 was retrieved from the Protein Data Bank (PDB ID 2w3l, Homo sapiens), subjected to cleaning, and prepared for docking. ROS, serving as the ligand, underwent structural definition, and molecular docking simulations were performed using AutoDock Vina [32] employing the Lamarckian genetic algorithm (LGA) within a specified search space encompassing the supposed Bcl-2 binding site. The search grid was centered at coordinates of center_x: 39.4679, center_y: 27.0653, and center_z: −12.3721, with dimensions of size_x: 40, size_y: 40, and size_z: 40. The calculation of binding free energy within the target receptor was focused on identifying the most valuable binding pose. Autodock was utilized to generate visual representations of the top-docked poses [33]. The docking pose with the lowest energy was selected for further analysis, and the interactions were examined using Discovery Studio v. 2.13.

### 2.7. Molecular Docking of ROS with β-CD

The most probable structure of the inclusion complex was determined by molecular docking studies using Autodock Tools 1.5.6 [34], employing the Lamarckian genetic algorithm (LGA). The three-dimensional structure of β-CD was obtained from the PDB file 3CGT, downloaded from the RCSB Protein Data Bank. The ligand (ROS) was prepared for docking by defining its rotatable bonds. The Autodock program was used, treating β-CD as a rigid receptor and the ROS molecule as a flexible ligand. Periodic boundary conditions were applied and energy minimization with a force tolerance was performed. The search grid for β-CD was centered at coordinates of center_x: 58.556, center_y: 11.883, and center_z: 8.870, with dimensions of size_x: 40, size_y: 40, and size_z: 40. All other parameters were set to their default values [35]. The program generated several feasible docked models for the most plausible structure of the β-CD inclusion complex based on energetic parameters.

### 2.8. Antioxidant Activity

An antioxidant capacity detection kit (ABTS method) was used to measure the total antioxidant capacity of ROS and ROS–β-CD. The prepared ABTS working solution was stored at room temperature in the dark for 12 h and then diluted 35 times with PBS to obtain the ABTS working solution. Next, 200 µL of the ABTS working solution was added to each well of a 96-well plate, followed by 10 µL of PBS, 10 µL of ROS (300 µM), and 10 µL of ROS–β-CD (300 µM). After incubation at room temperature for 3 min, the OD value at 734 nm was measured using a microplate reader.

### 2.9. In Vitro Cytotoxicity

The main objective of this experiment was to assess the in vitro cytotoxicity of ROS and ROS–β-CD in MD-MBA-231 breast cancer cell lines using the CCK8 assay [36]. A total of 5 × 10^3^ cells/well MD-MBA-231 were seeded in 96-well plates and allowed to incubate for 24 h. Subsequently, various concentrations of ROS and ROS–β-CD (ranging from 200 to 400 µM) suspended in culture medium were introduced. After an additional 24 h of incubation, 10% CCK8 solution was added and absorbance was measured at 450 nm. Cell viability was determined using a standard formula.

### 2.10. Cellular Uptake

A confocal microscope was used to monitor the cellular uptake at a series of time points (0.5, 3, 5, and 12 h) in MD-MBA-231 cells with different durations of incubation. Flow cytometry was also run to quantify the cellular uptake of RhoB within the same incubation times.

### 2.11. Cell Apoptosis Assay

The primary aim of this investigation was to assess the impact of ROS and ROS–β-CD on cell apoptosis utilizing an annexin V–FITC/PI kit. To achieve this, MD-MBA-231 breast cancer cells were seeded in 6-well plates at a density of 1 × 10^6^ cells/well and exposed to a concentration of 300 µM of the compounds (ROS and ROS–β-CD) for 24 h. Following treatment, cells were collected, washed with PBS, and subjected to staining using an annexin V–FITC/PI apoptosis assay kit. The proportion of apoptotic cells was then quantified using CytoFLEX flow cytometry [37].

### 2.12. Three-Dimensional (3D) Tumor Spheroid Analysis

The MDA-MB-231 cells were seeded in a 96-well plate containing 200 μL of the medium. Tumor spheroids were cultured and stored in a cell culture incubator at 37 °C in a 5% carbon dioxide atmosphere and the medium changed every two days to maintain the growth of tumor spheroids. After 2 days, tumor spheroids had formed, which were then treated with ROS and ROS–β-CD and (300 μM) for 48 h. Images are taken with a confocal microscope (DLS, Leica, Germany).

### 2.13. Western Blot

MD-MBA-231 cells were cultured in six-well plates at a seeding density of 1 × 10^6^ cells per well for 24 h. Subsequently, the cells were treated with 300 µM of ROS and ROS–β-CD for an additional 24 h. Following treatment, the cells were harvested and lysed on ice for 30 min in the presence of protease inhibitors. The lysate was then subjected to centrifugation at 12,000 rpm for 10 min at 4 °C, and the resulting supernatant was quantified using a protein assay kit obtained from Yeasen Bio., Shanghai, China. The proteins were separated on a 10% Bis-Tris polyacrylamide gel obtained from Beyotime, Nantong, China, and subsequently transferred onto a PVDF membrane from BioRad, Hercules, CA, USA. The membrane was then blocked using 5% skim milk powder and incubated overnight with primary antibodies. Following the overnight incubation, protein expression levels were assessed using ECL chemiluminescence from Beyotime, Nantong, China, with an appropriate secondary antibody sourced from Yeasen Bio. Quantitative analysis of protein relative expression was achieved using Image J software 9.0 [38].

## 3. Results and Discussion

### 3.1. Preparation and Characterization of ROS–β-CD

**Figure 1:**
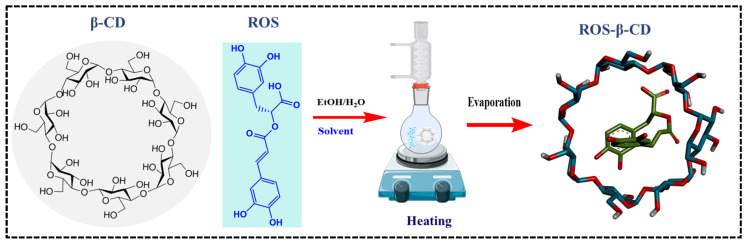
Graphical illustration of the mechanism of the encapsulation of ROS in the cavity of β-CD, leading to the formation of the host–guest ROS–β-CD inclusion complex.

**Figure 2:**
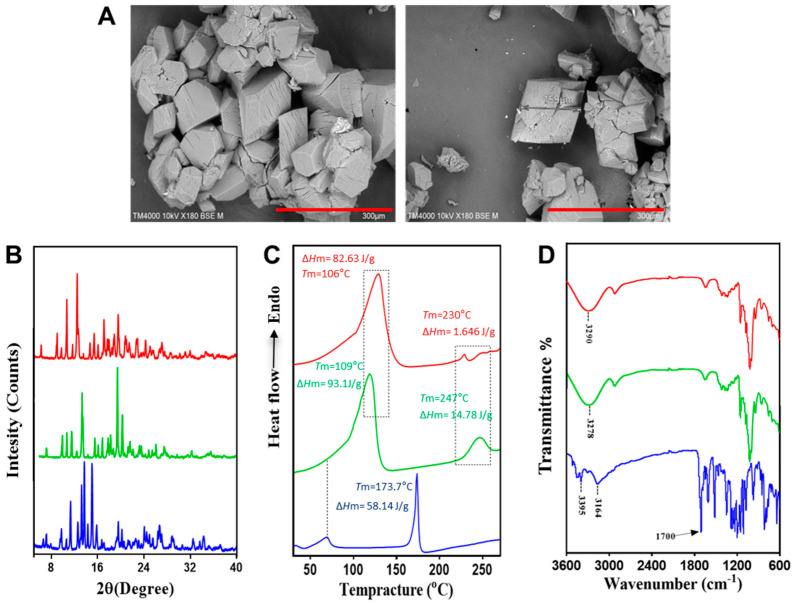
Solid-state characterization of ROS–β-CD: (**A**) SEM images, scale bar = 300 µm, (**B**) powder XRD, (**C**) DSC, and (**D**) FT-IR analysis. Color legends: blue (ROS), red (β-CD), and green (ROS–β-CD).

The preparation and characterization of the ROS–β-CD inclusion complex, formed by the interaction between ROS and β-CD, involve particular procedures for successful complexation. Equal molar ratios of ROS and β-CD were used in optimizing the formation of the inclusion complex. The components undergo dissolution in an appropriate solvent, often employing a co-solvent system to enhance solubility and facilitate the complexation process [39]. The solution is subjected to stirring to enhance the interaction between ROS and β-CD (Figure 1). Upon crystallization at optimized conditions, the white candy-shaped crystalline form (Figure 2A) of ROS–β-CD was obtained via the solvent evaporation method. The obtained ROS–β-CD crystals were subjected to further characterization.

### 3.2. Powder X-Ray Diffraction (PXRD)

Powder X-ray diffraction (PXRD) is an essential technique for the comprehensive characterization of inclusion complexes, particularly in the context of crystalline substances [40]. Inclusion complexes represent the formation of host–guest molecular associations, wherein a designated molecule (the guest) becomes integrated within the crystalline lattice of another molecule (the host). PXRD serves as an invaluable tool, providing a vital understanding of the compositional constituents, and crystalline properties essential to these complexes. The PXRD patterns of ROS, β-CD, and ROS–β-CD are shown in Figure 2B. The inclusion complex ROS–β-CD exhibited characteristic peak values at 2θ (6.22°, 9.10°, 9.86°, 10.72°, 11.56°, 12.58°, 13.10°, 14.82°, 15.42°, 16.18°, 17.16°, 17.22°, 17.72°, 18.86° and 19.73°). Many characteristic peaks of the ROS and β-CD shifted compared with ROS–β-CD, indicating the successful formation of the inclusion complex.

### 3.3. Differential Scanning Calorimetry (DSC)

Differential scanning calorimetry (DSC) is vital in the analysis and understanding of inclusion complexes. A thermal analysis technique, DSC quantifies the heat flow associated with physical and chemical modifications within a sampling across a temperature gradient [41]. Hence, DSC stands as a vital technique for analyzing the thermodynamic and thermal attributes of inclusion complexes. The DSC analyses of ROS, β-CD, and ROS–β-CD are shown in Figure 2C. In the DSC analysis, the newly synthesized ROS–β-CD inclusion complex has different melting points when compared with ROS and β-CD. ROS indicated its anhydrous crystalline state, exhibiting a sharp endothermal effect at 176 °C. β-CD showed a broad endothermal peak at 106 °C and a short endothermal peak at 230 °C. In comparison, the endothermal peak of ROS–β-CD was nearly comparable with β-CD, with a slight increase and decreased endothermal effect compared with ROS having new endothermal effects at 109 °C and 247 °C. These changes in the thermal behavior indicated the formation of the ROS–β-CD inclusion complex.

### 3.4. Fourier Transform Infrared Spectroscopy (FT-IR)

Fourier transform infrared spectroscopy (FT-IR) plays a key role in investigating inclusion complexes, providing valuable insights into their structural and molecular characteristics [42]. This analytical technique enables the identification of specific functional groups within both the host and guest molecules of an inclusion complex, as revealed by distinctive absorption bands corresponding to various chemical moieties [43]. Changes in the FT-IR spectra, including shifts in peak positions, the emergence of new peaks, or alterations in peak intensities, serve as indicators of complex formation, offering evidence of interactions between the host and guest molecules. Moreover, FT-IR spectra provide valuable insights into the nature and strength of these interactions, allowing the inference of hydrogen bonding, van der Waals forces, and other intermolecular interactions based on spectral changes. Figure 2D presents the FT-IR analysis of ROS, β-CD, and ROS–β-CD. The FT-IR spectrum for ROS–β-CD seems nearly like the pure CDs observed by other researchers [21]. The FT-IR spectrum of ROS revealed a broad absorption band at 3181 cm^−1^, indicative of the carboxylic acid hydroxyl (OH) group with a broad profile. Additionally, distinct bands were observed at 1718 cm^−1^ corresponding to the carbonyl group C=O of the carboxylic acid, 1683 cm^1^ representing the carbonyl group C=O conjugated with a double bond, and 1514 and 1481 cm^−1^ associated with the stretching of the aromatic ring. Furthermore, the range of 1400–1350 cm^−1^ displayed signals related to the stretching of the C-O-C bonds, while the β-CD exhibited prominent peaks at 3370, 2928, 1157, and 1029 cm^−1^, corresponding to the symmetric and anti-symmetric stretching of OH, CH_2_, and C–C and the bending vibration of the O–H functional group. The broad hydroxyl band observed at 3290 cm^−1^ in the FT-IR spectrum of pure CD exhibited a significant narrowing and shift to 3278 cm^−1^ in the spectrum of the ROS–β-CD inclusion complex. This narrowing stands as a favorable sign indicating the successful formation of the inclusion complex, a phenomenon characteristically observed by researchers in the synthesis of inclusion complexes that involve both host and guest molecules. The absorption peak at 3181 cm^−1^ of the (OH) and distinct bands at 1718 cm^−1^ corresponding to the carbonyl group C=O of the carboxylic acid of ROS disappeared in the spectrum of ROS–β-CD, indicating that these two functional groups are securely encapsulated in the cavity of the CD molecule. The alteration in frequency observed between the constituent molecule and its inclusion complex may attributed to modifications in the microenvironment. These changes result in the formation of hydrogen bonding and the involvement of van der Waals forces during their interaction, facilitating the formation of the inclusion complex.

### 3.5. Solubility and Dissolution Rate

**Figure 3:**
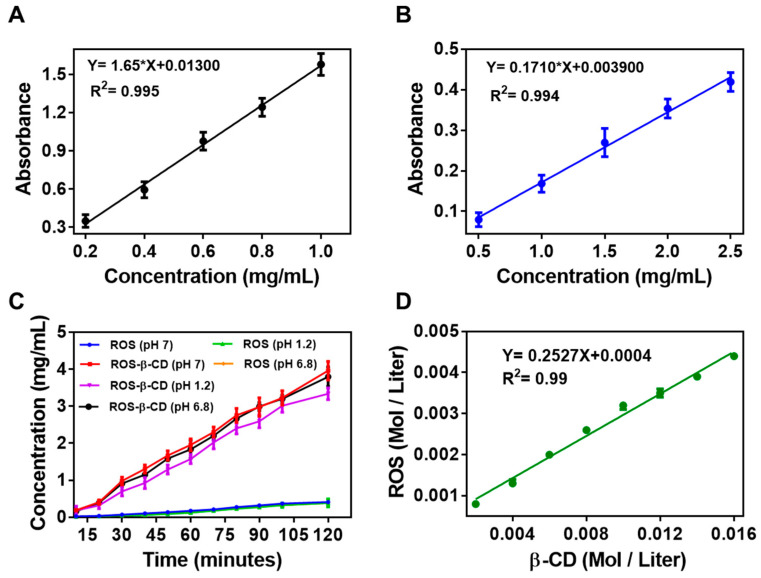
(**A**) Standard curve of ROS, (**B**) standard curve of ROS–β-CD, (**C**) dissolution rate, (**D**) phase solubility diagram of ROS dependence on the concentration (mM) of CD.

The solubility of drugs holds significant importance in the pharmaceutical field, impacting the bioavailability of drugs. High solubility facilitates rapid dissolution, whereas low solubility poses challenges to drug absorption and effectiveness. Both solubility and dissolution rate are pivotal considerations in the pharmaceutical formulation and design process to optimize the therapeutic efficacy of drugs [44]. The solubility and dissolution rate of ROS and ROS–β-CD were determined using a standard curve (Figure 3A,B), which was plotted by measuring the absorbance of different concentrations at their λ_max_. Notably, ROS–β-CD exhibited heightened solubility (16.8 mg/mL) in comparison to free ROS (1 ≥ mg/mL). Additionally, the dissolution rate of ROS–β-CD surpassed that of ROS, as depicted in Figure 3C. The remarkable time-dependent dissolution rate in various buffers for simulating in vivo environments with different pH values (pH 7, acidic medium pH 1.2 and phosphate buffer pH 6.8) observed in ROS–β-CD can be ascribed to the abundance of hydrogen bonding interactions, fostering competitive interactions with the solution medium and facilitating the dissociation of the subcomponents. Consequently, the formulation of ROS–β-CD effectively enhanced the solubility and dissolution rate of ROS, potentially enhancing its bioavailability and therapeutic efficacy.

### 3.6. Phase Solubility Calibration

The phase solubility of ROS in the presence of β-CD determines the impact of varying β-CD concentrations on ROS solubility [45]. This study sheds light on β-CD capacity as a solubilizing agent for hydrophobic compounds, addressing the challenge posed by the limited aqueous solubility of ROS. We systematically assessed ROS phase solubility under various concentrations of β-CD, aiming to gain insights into complex formation and conditions conducive to enhanced solubility. The observed phase solubility pattern, classified as A_L_-type (Figure 3D), suggests the formation of the complex between ROS and β-CD in a stoichiometry of 1:1. The apparent binding constant (Kc) was determined to be 143.28 M⁻^1^, which is nearly comparable with that previously reported in [25], while the complexation efficiency (CE) was found to be 0.33. The nature of solubility increase, whether linear or saturable, indicates favorable interactions between ROS and β-CD. The findings offer valuable insights into β-CD potential as a solubilizing agent for ROS, with the observed behavior suggesting the formation of a stable complex. Hence, β-CD emerges as a promising candidate for enhancing the aqueous solubility of ROS.

### 3.7. Molecular Docking

**Figure 4:**
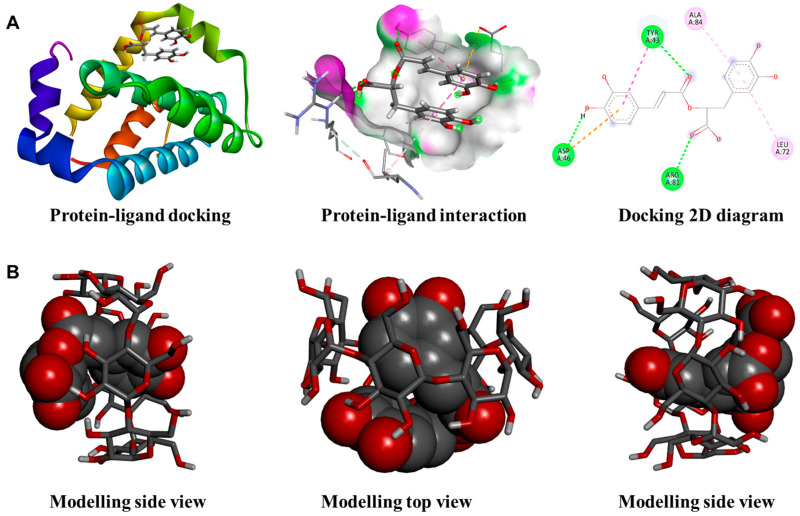
Molecular docking study of (**A**) ligand (ROS)–protein (Bcl-2) docking with 2D visualization, generated via Discovery Studio, shows the interaction between the ligand and target protein residues and (**B**) optimized structure of ROS–β-CD inclusion complex formations. The optimized structure was generated through Mercury 4.0. The visual representation of ROS–β-CD contains ROS molecules in space filling while β-CD is in the stick mode.

Rosmarinic acid (ROS) is utilized in the treatment of various cancers due to its ability to act through multiple pathways, including inhibition of tumor cell proliferation, induction of apoptosis, suppression of metastasis, and reduction in inflammation [46]. It induces apoptosis and decreases the Bcl-2 ratio primarily via mitochondrial apoptotic pathways in osteosarcoma cells. Additionally, in breast cancer, it enhances caspase cleavage and reduces the expression and protein levels of Bcl-2 following ROS treatment [47]. In silico-based approaches have offered a novel perspective on the development of highly efficient chemical leads for creating new formulations targeting cancer cells [48]. To rationalize the promising anticancer activity obtained by ROS, a molecular docking study was performed to support the mode of action, interaction, and preferred binding sites of the targeted compound (ROS) with the active sites of the Bcl-2 protein. Molecular docking of ROS (as ligand) with Bcl-2 (target protein) was performed, as shown in Figure 4A. From the ensuing docked structures, it is clear that the ROS molecules can easily fit into the active site of the Bcl-2 protein receptor. The lowest energy-docked (−7.5 kcal/mol) conformation is considered indicative of efficient binding, representing the energy required for a ligand to interact with the entire surface of a protein. This conformation of the targeted candidates signifies efficient binding, highlighting the energy necessary for complete surface coverage of the enzyme by the ligand. The molecular docking interactions of ROS with the target protein reveal vigorous conventional hydrogen bonding interactions with TYR_A:43, ASP_A:46, and ARG_A:41 residue of the target protein. Another type of short contact, such as *pi* anion (ASP A: 46), *pi*–*pi* stacked (TYR_A: 43), and *pi* alkyl (LEU_A:72, ALA_A:84), of ROS with target protein residues exist. The potent interactions between the ROS and target protein residues facilitate their deep penetration into the receptor protein cavity, serving as anticancer agents to inhibit cancer cell growth.

### 3.8. Molecular Modeling

Molecular modeling is vital for enhancing experimental analyses of inclusion complexes, providing a detailed understanding of molecular-level intricacies. This computational approach involves assessing and simulating the structural and dynamic properties of complexes formed between molecules, specifically within the host–guest inclusion complex. In the context of the ROS–β-CD inclusion complex, molecular modeling techniques are employed to predict the spatial arrangement of molecules within the complex. This not only enhances experimental investigations but also contributes to the development of innovative drug design and delivery strategies, offering insights into the energetics, stability, and conformational aspects of the inclusion complex. Molecular docking studies using Autodock Tools 1.5.6 determined the inclusion complex’s most probable structure. Docking studies were conducted using AutoDock Vina to investigate the binding affinity between ROS and β-CD. The ligand (ROS) successfully docked into the binding pocket of β-CD, and the most probable conformation is illustrated in Figure 4B and Supplementary Figure S4. The results indicated a 1:1 ratio for the effective combination of the ROS with β-CD. The inclusion complex exhibited an estimated free energy of binding at −6.4 kcal/mol for the best pose. Molecular modeling results unveiled that the guest molecules fit into the hydrophobic host cavity, adopting a bent conformation, and thus supporting the host–guest inclusion complex of ROS–β-CD.

### 3.9. Antioxidant Activity

Antioxidant activity plays a critical role in neutralizing reactive oxygen species (ROS), thereby protecting cells from oxidative damage, reducing the risk of chronic diseases, delaying the aging process, enhancing immune function, and contributing to overall health and longevity. To assess the antioxidant potential of ROS and ROS–β-CD, we utilized a total antioxidant capacity assay kit (ABTS method) from Beyotime. The measured average antioxidant capacities were: PBS: −0.09 mM, ROS: 0.32 mM, and ROS–β-CD: 0.48 mM (Supplementary Figure S5). These results demonstrate that ROS–β-CD exhibits significantly higher total antioxidant capacity compared to free ROS.

### 3.10. In Vitro Cytotoxic Effect

**Figure 5:**
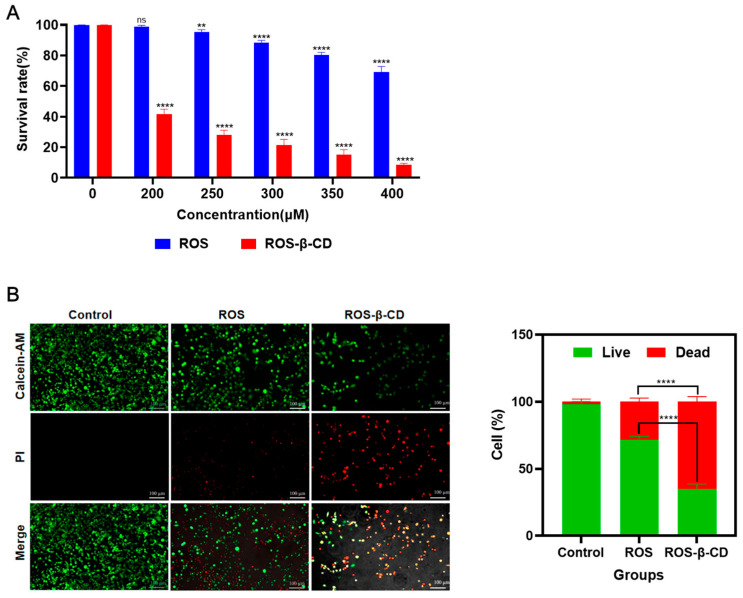
Cytotoxic effects on MDA-MB-231 cells. (**A**) MDA-MB-231 cells were treated with 200–400 μM of ROS and ROS–β-CD for 48 h; (**B**) live–dead cell images of MDA-MB-231 cells with different treatments, stained with calcein AM (green, viable) and PI (red, dead). Statistical significance between was calculated via ANOVA: ** *p* < 0.01, and **** *p* < 0.0001, while “ns” is not significant.

Cyclodextrins (CDs) are exceptional biocompatible drug carriers, exhibiting low toxicity and the ability to form water-soluble inclusion complexes with pharmaceutical drugs [49]. They are widely employed to enhance drug bioavailability, and encapsulating drugs within CDs has been shown to improve cytotoxicity [50]. Drugs loaded with CDs and other delivery systems, where the active compounds occupy the empty cavities of CDs, demonstrate cytotoxicity primarily due to the increased permeability and uptake of these drugs, rather than any direct effects of the CDs themselves [51]. To assess cytotoxicity, MB-MDA-231 cells endured exposure to various concentrations of ROS and ROS–β-CD during a 48 h incubation period, as illustrated in Figure 5A. The free ROS exhibited slight toxicity towards MB-MDA-231 cells, ascribed to the elevated concentration needed for a 50% reduction in cell viability. Conversely, the ROS–β-CD inclusion complex demonstrated heightened and dose-dependent cytotoxicity. Remarkably, ROS–β-CD exhibited superior cytotoxicity compared to free ROS. Additionally, we performed a live–dead staining experiment, which showed that ROS–β-CD treatment had stronger red fluorescence and also demonstrated that ROS–β-CD is more cytotoxic compared to free ROS, as shown in Figure 5B. This enhanced effect is likely attributable to the presence of β-CD facilitating the uptake of the ROS–β-CD inclusion complex by tumor cells (Supplementary Figure S7). This intriguing outcome highlights the potential of β-CD as a biocompatible co-former to enhance drug absorption, resulting in increased anticancer efficacy against MB-MDA-231 cancer cells, potentially leading to enhanced biopharmaceutical performance.

### 3.11. Cell Apoptosis Assay

**Figure 6:**
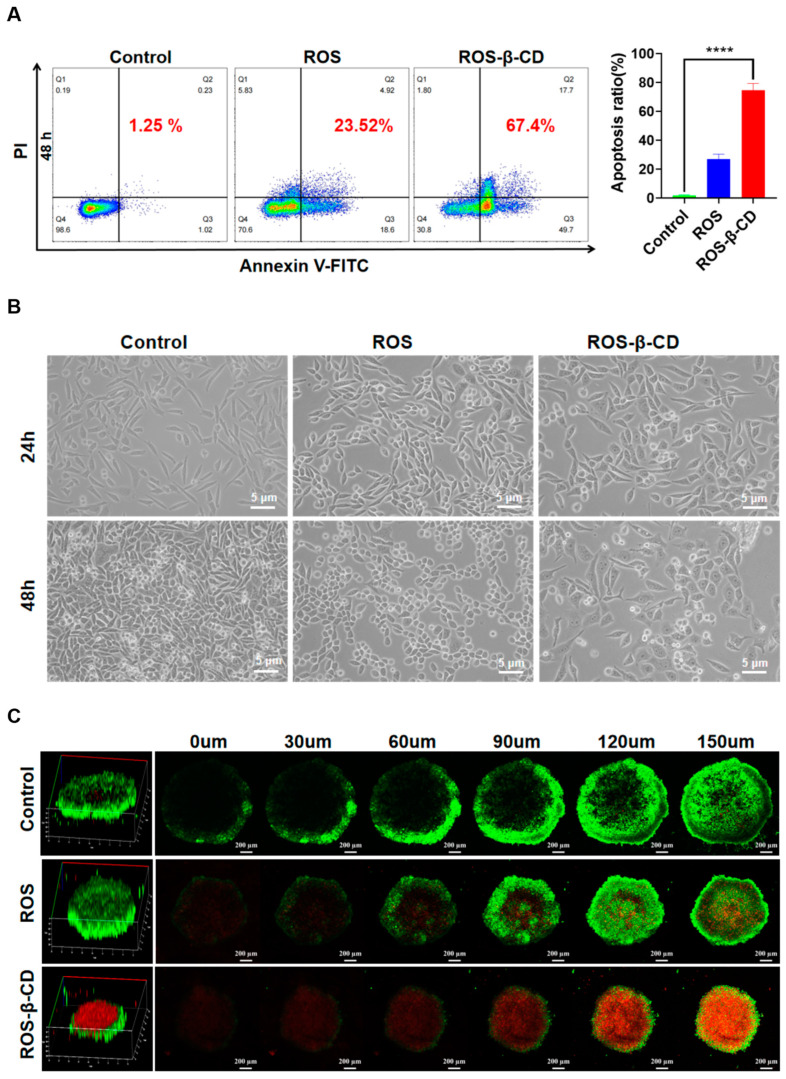
In vitro analysis of apoptosis, morphology, and 3D cell spheroids of MDA-MB-231 cells treated with or without ROS. (**A**) Apoptotic effect on MDA-MB-231 cells, (**B**) morphological image of MDA-MB-231 cells treated with free ROS and ROS–β-CD, Scale bar = 5 µm. (**C**) representative CLSM images of 3D cell spheroids (treated cells), stained with calcein AM (green, alive) and PI (red, dead). Statistical significance between groups was calculated via ANOVA: **** *p* < 0.0001. Scale bar = 200 µm.

The apoptosis analysis by flow cytometry was performed using the annexin V–FITC/PI dual-staining method to further understand the growth inhibition mechanism of ROS–β-CD-treated MDA-MB-231 cells [52,53]. An annexin V–FITC staining chart consists of four quadrants (O), where Q1 indicates necrotic cells, Q2 and Q3 represent late and early apoptotic cells, respectively, and Q4 is viable cells. We used untreated negative control live cells and a positive apoptosis control (Supplementary Figure S8) in cells treated with hydrogen peroxide, which induces apoptosis. Figure 6A shows a significant apoptotic effect after 48 h of co-incubation with MDA-MB-231 cells with ROS–β-CD at a concentration of 300 μM. Under the experimental concentrations, free ROS exhibited slight cytotoxicity, with a 23.53% apoptosis rate on MDA-MB-231 cells. In contrast, ROS–β-CD displayed an elevated 67.4% apoptosis rate on MDA-MB-231 cells. Compared to the ROS treatment, the ROS–β-CD inclusion complex exhibited almost a threefold (43.9%) higher apoptotic rate. These results are consistent with the observations made in the cell viability assays, indicating the heightened effectiveness of ROS–β-CD.

### 3.12. Cell Morphology

To confirm the results obtained with the CCK8 method, MDA-MB-231 cells were treated with ROS and ROS–β-CD (300 μM) for 24 h and 48 h and observed under electron microscopy. As shown in Figure 6B, compared with the control, the morphology of MDA-MB-231 cells treated with ROS–β-CD 300 μM for 24 h had shrunk, the cell membrane was ruptured, and almost all cells had died at 48 h. However, with the same concentration of ROS, the cells increased and gradually became rounded, suggesting that ROS had a slight effect on cell viability [47]. Hence, ROS–β-CD can inhibit the proliferation of breast cancer cells and kill breast cancer cells.

### 3.13. In Vitro 3D Tumor Spheroid Modeling Antitumor Effect

The antitumor activity of ROS and ROS–β-CD was further investigated using 3D tumor spheroids. The three-dimensional tumor spheroids accurately mimic tumor environments, facilitating investigation into architectural, cellular, and treatment dynamics, and advancing cancer therapy research. Compared to traditional two-dimensional monolayer structures, three-dimensional tumor spheroids can more accurately simulate in vivo therapeutic effects, allowing for more rapid and cost-effective preclinical drug testing of anticancer drugs [54]. The results showed (Figure 6C and Supplementary Figure S9) that the most dead cells (red) were found in ROS–β-CD-treated 3D tumor spheroids. These three-dimensional tumor spheroids offer a physiologically relevant model, improving drug testing accuracy and aiding in discovering new therapies.

While 3D tumor spheroids provide a more physiologically relevant model compared to traditional 2D cultures by more accurately mimicking the tumor microenvironment, they still have limitations. Despite their utility for preliminary screening, 3D spheroids cannot fully replicate the complexity of in vivo models, which are crucial for a thorough assessment of treatment efficacy and safety prior to clinical application. Future research may use in vivo studies to further validate the efficacy and safety of the ROS–β-CD complex, elucidating the molecular mechanisms responsible for its enhanced anticancer activity, developing advanced pharmaceutical formulations to optimize delivery, and advancing clinical trials to translate these findings into effective therapeutic strategies for TNBC.

### 3.14. Western Blotting

**Figure 7:**
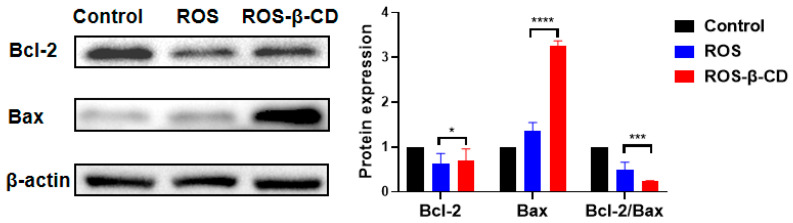
Representative Western blot image and quantitative analysis of relative protein expression. MDA-MB-231 cells were treated with ROS–β-CD and ROS for 48 h. ANOVA was used to calculate the differences between the treatment groups. * *p* < 0.05, *** *p* < 0.001, and **** *p* < 0.0001 indicate statistically significant differences.

Bcl2 and Bax are pivotal regulatory proteins within the Bcl-2 family, with Bcl2 acting as an inhibitor and Bax as an inducer of apoptosis. Modulating Bcl-2 downregulation or Bax upregulation has been linked to enhanced apoptosis across various tumor cell types. The Bcl-2/Bax expression ratio within tumors is closely associated with tumor initiation and progression. Western blot analysis was conducted to assess the influence of ROS and ROS–β-CD on the expression levels of these two key proteins, as depicted in Figure 7. In MDA-MB-231 cells exposed to treatment with 300 μM ROS and the ROS–β-CD inclusion complex, the ROS–β-CD group exhibited a notable decrease in Bcl2 protein expression compared to the ROS group, accompanied by a significant increase in Bax protein expression and a reduced Bcl2/Bax ratio (*p* < 0.001). These findings suggest that both ROS–β-CD and ROS contribute to the induction of tumor cell apoptosis. Furthermore, ROS–β-CD demonstrated superior efficacy in inducing cell apoptosis, highlighting the potential of the ROS–β-CD inclusion complex as a promising therapeutic agent.

## 4. Conclusions

The host–guest inclusion complex ROS–β-CD exhibits considerable potential for pharmaceutical applications, particularly by addressing the solubility and dissolution limitations of ROS. Characterization studies confirmed a 1:1 stoichiometry and a stable inclusion mode driven by non-covalent interactions, resulting in a marked increase in solubility and dissolution rate. These physicochemical enhancements are directly linked to the improved anticancer efficacy observed in vitro. The inclusion complex displayed potent dose- and time-dependent cytotoxic effects against TNBC cells, highlighting the critical role of enhanced ROS bioavailability in its therapeutic performance. These results contribute significantly to the field of pharmaceutical sciences, reinforcing the value of host–guest complexation as an approach for improving bioactive compound performance. The research thus provides a robust framework for future drug development, effectively connecting characterization insights with practical anticancer applications.

## Data Availability

The dataset used throughout this work is available upon request.

## Supplementary Materials

The following supporting information can be downloaded at: https://www.mdpi.com/article/10.3390/pharmaceutics16111408/s1, Figure S1. UV spectra of ROS and ROS-ß-CD. Figure S2. Stability of ROS–β-CD at different pH. Figure S3. Stability of ROS–β-CD in dark and light. Figure S4. Optimized geometrical structure of ROS–β-CD. Figure S5. Antioxidant activity. Figure S6. SEM images of pure rhodamine b (RhoB) and its inclusion complex with β-CD (β-CD@RhoB). Figure S7. Confocal microscopy images and flow cytometry diagram showing the cellular uptake of rhodamine b (RhoB) and its inclusion complex with β-CD (β-CD@RhoB) within different periods of incubation. Figure S8. Positive apoptosis control in cells treated with hydrogen peroxide. Figure S9. CLSM images of 3D cell spheroids with different treatments s, stained with calcein AM (green, alive) and PI (red, dead).
