# Supplementary material for: Optimizing Antitumor Effect of Triple-Negative Breast Cancer via Rosmarinic Acid–β-Cyclodextrin Inclusion Complex
Source: Pharmaceutics. 2024 Nov 1;16(11):1408. doi: 10.3390/pharmaceutics16111408 (PMC11597731; doi:10.3390/pharmaceutics16111408)
Supplement: Supplementary file 1 [file pharmaceutics-16-01408-s001.zip › pharmaceutics-3225122-supplementary.pdf]

Optimizing Antitumor Effect of Triple Negative Breast Cancer via Rosmarinic Acid-β-Cyclodextrin Inclusion Complex

Yuan Li[1, 2†], Muhammad Inam[1, 2, 3†], Muhammad Waqqas Hasan[1, 2], Kaixin Chen[4], Zhongqian Zhang[1, 2], Yongcheng Zhu[1], Jiayu Huang[1], Zhuowen Wu[1,], Wenjie Chen [1, 2, 5*,] Min Li [1, *]

1. Department of Emergency, the Second Affiliated Hospital, Guangzhou Medical University, Guangzhou 510260, China
2. Guangdong Province & NMPA & State Key Laboratory, School of Pharmaceutical Sciences, Guangzhou Medical University, Guangzhou 511436, China
3. Medical Science and Technology Innovation Center, School of Chemistry and Pharmaceutical Engineering, Shandong First Medical University and Shandong Academy of Medical Sciences, Jinan 250000, China
4. Graduate School of Biomedical Engineering, ARC Centre of Excellence in Nanoscale Biophotonics, Faculty of Engineering, UNSW Sydney, NSW 2052, Australia
5. Sydney Vital Translational Cancer Research Centre, Westbourne St, NSW 2065, Australia

[†:] These authors contributed equally to this work

Corresponding authors: wenjie.chen1@hdr.mq.edu.au, 2008690617@gzhmu.edu.cn

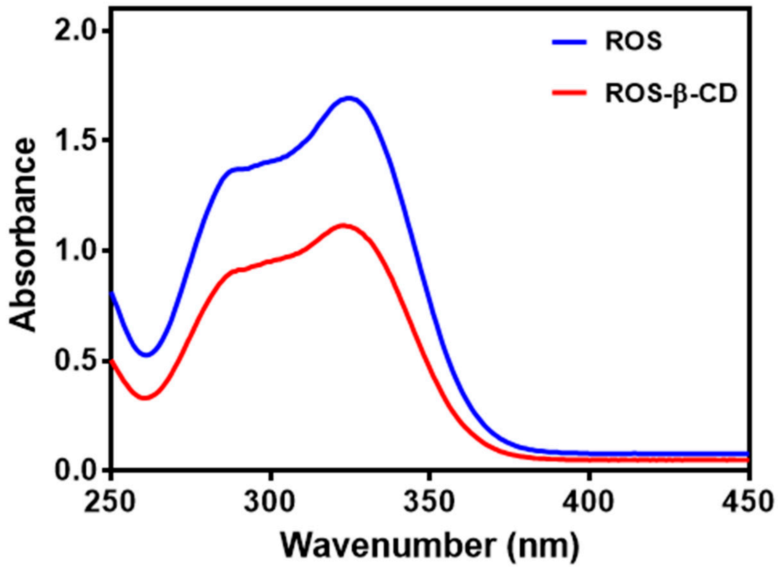

Figure S1. The UV spectra of ROS and ROS-β-CD

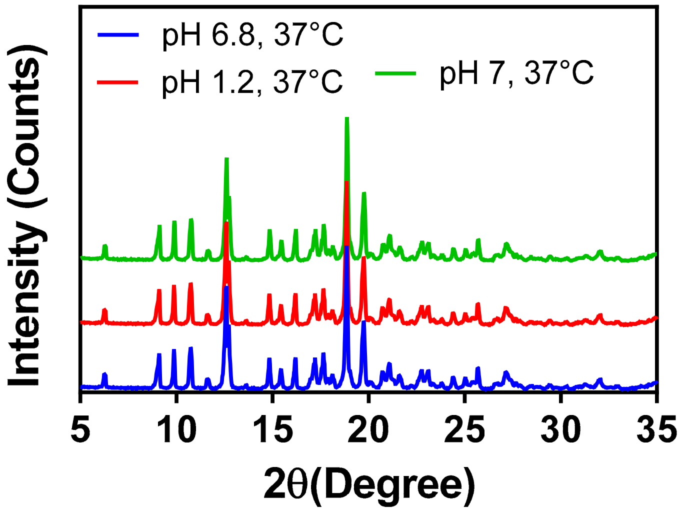

Figure S2. Stability of ROS-ß-CD at different pH

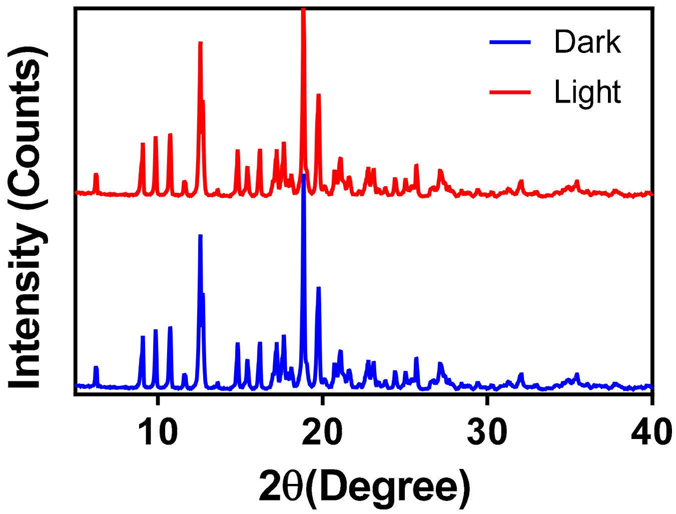

Figure S3. Stability of ROS-ß-CD in dark and light

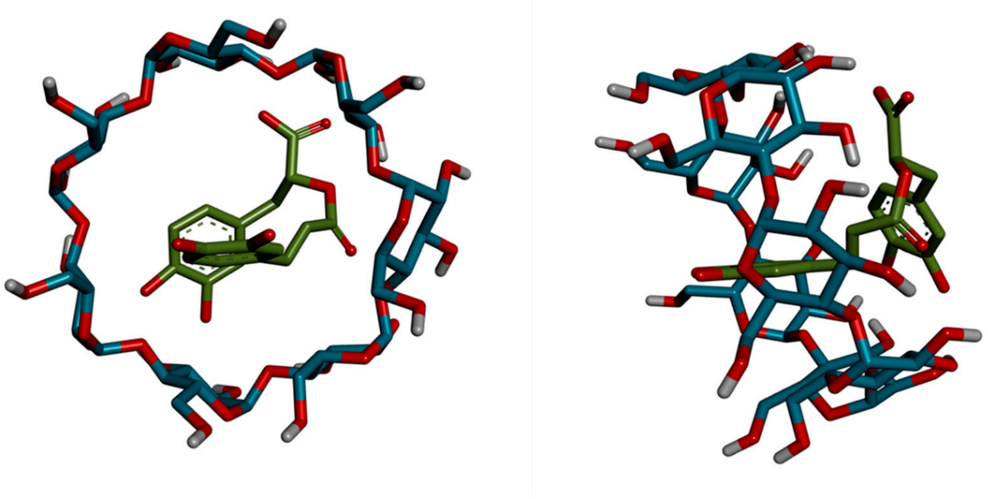

Figure S4. The optimized geomatical structure of ROS-β-CD

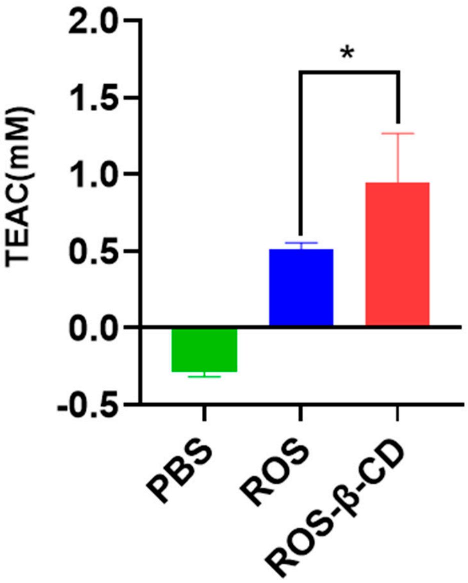

Figure S5. The antioxidant activity

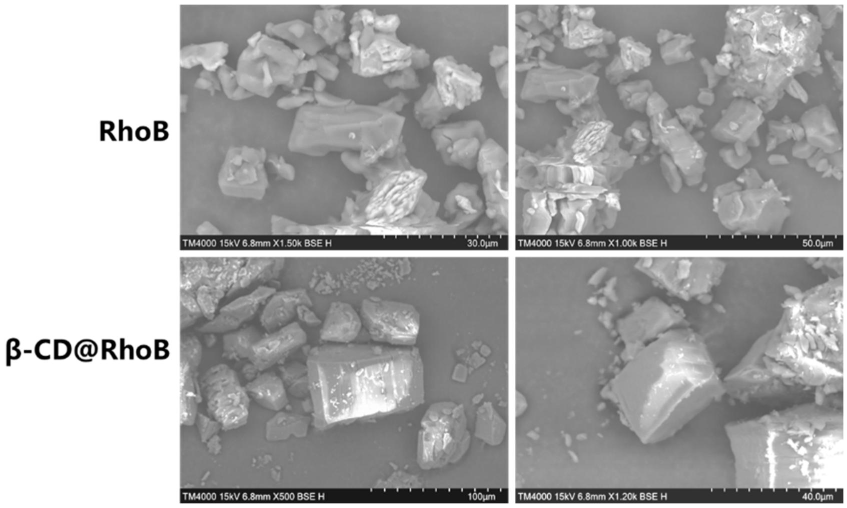

Figure S6. The SEM images of pure rhodamine b (RhoB) and its inclusion complex with β-CD (β-CD@RhoB).

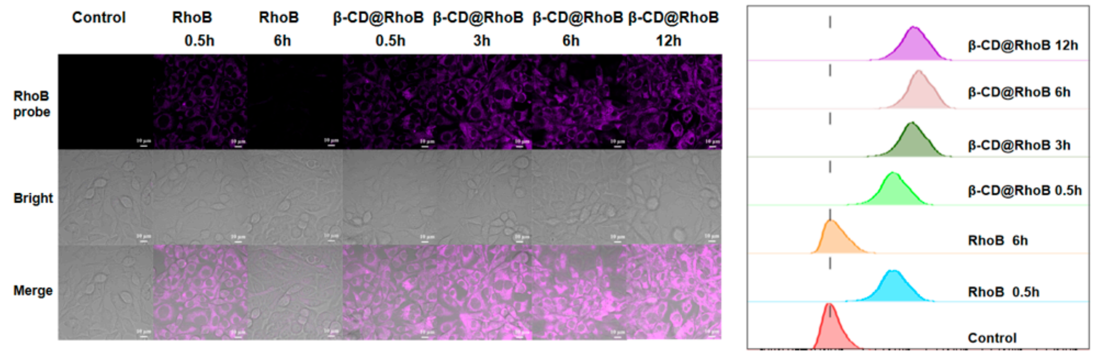

Figure S7. The confocal microscopy images and flow cytometry diagram showing the cellular uptake of rhodamine b (RhoB) and its inclusion complex with β-CD (β-CD@RhoB within different time of incubation.

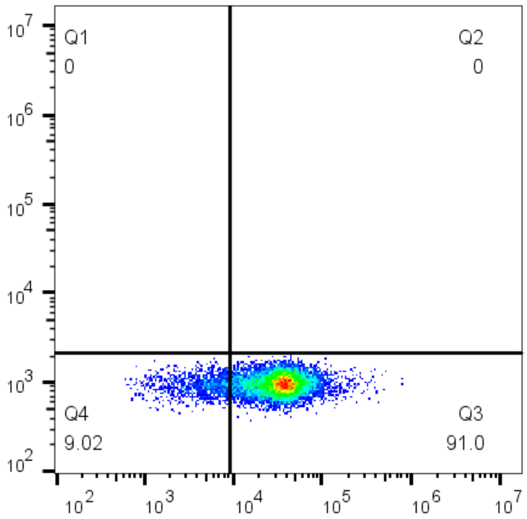

Figure S8. Positive apoptosis control in cells treated with hydrogen peroxide.

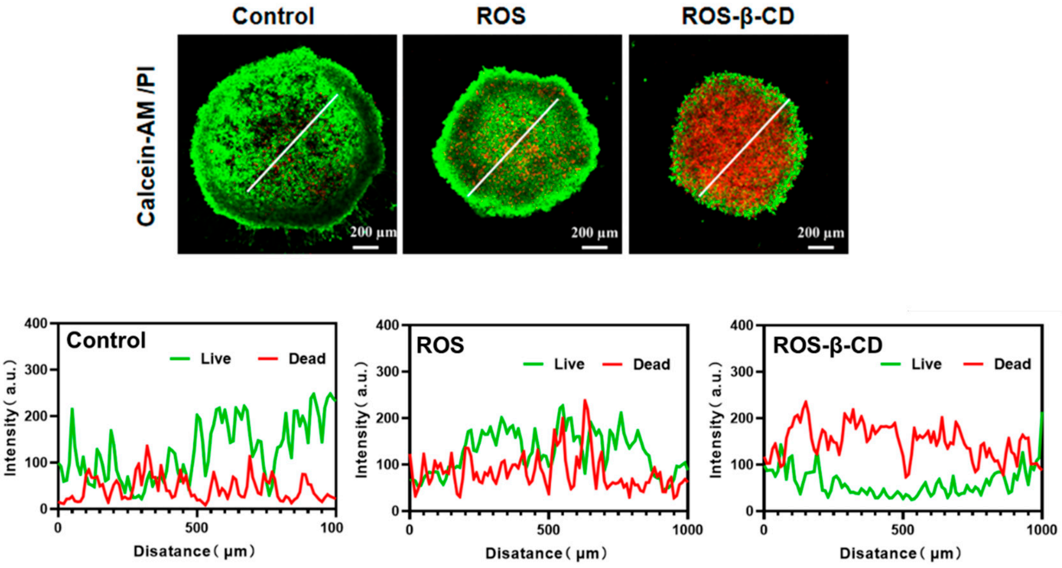

Figure S9. CLSM images of 3-D spheroids treated cells stained with Calcein-AM (green, alive) and PI (red, dead) and the line profile indicating the colocalization analysis of these two channels using ImagJ.

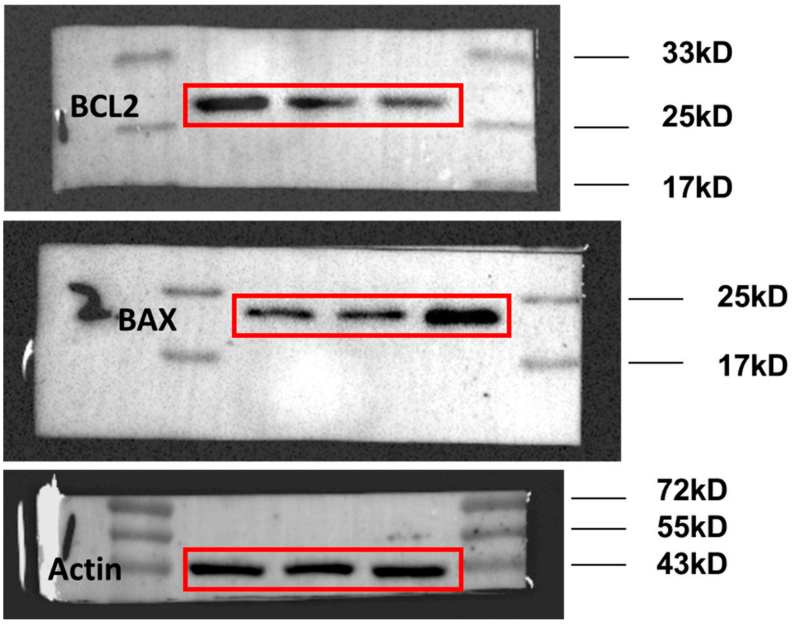

Source image of WB of **Figure 7**
